# Chinese Tone and Vowel Processing Exhibits Distinctive Temporal Characteristics: An Electrophysiological Perspective from Classical Chinese Poem Processing

**DOI:** 10.1371/journal.pone.0085683

**Published:** 2014-01-09

**Authors:** Weijun Li, Lin Wang, Yufang Yang

**Affiliations:** Key Laboratory of Behavioral Science, Institute of Psychology, Chinese Academy of Sciences, Beijing, China; Vanderbilt University, United States of America

## Abstract

Classical Chinese poems have strict regulations on the acoustic pattern of each syllable and are semantically meaningless. Using such poems, this study characterized the temporal order of tone and vowel processing using event-related potentials (ERPs). The target syllable of the poem was either correct or deviated from the correct syllable at tone, vowel or both levels. Vowel violation elicited a negative effect between 300 and 500 ms regardless of the tone correctness, while tone violation elicited a positive effect between 600 and 1000 ms. The results suggest that the vowel information was available earlier than the tone information. Moreover, there was an interaction between the effect of vowel and tone violations between 600 and 1000 ms, showing that the vowel violation produced a positive effect only when the tone was correct. This indicates that vowel and tone processing interacts in the later processing stage, which involves both error detection and reanalysis of the spoken input. Implications of the present results for models of speech perception are discussed.

## Introduction

In Mandarin Chinese, each syllable comprises of three components, namely, initial sound, final sound and tone. The initial sound is normally a consonant, while the final sound generally contains at least one vowel. There are six vowels in Chinese, including /a/, /o/, /e/, /i/, /u/, /ü/, while the primary vowel refers to the most important vowel when there is more than one vowel in the final sound. The variation of tone (high, low, rising, and falling) is mainly carried by the final sound, and is marked on the primary vowel [1]. Both consonant and vowel belong to segmental information, because they can be separated and occur in a distinct temporal order. Tone, on the other hand, belongs to supra-segmental information, since it normally coexists with segmental information and cannot be discretely ordered with them. Although tonal and segmental units are an integral part of Chinese syllable structure, they are taken as independent phonetic units [2], [3], [4]. Listeners have to rely on tonal information to distinguish the meaning of Chinese syllables except for segmental information.

A number of studies have attempted to investigate the processing differences between tonal and segmental information during speech perception. It was generally found that the segmental information had “perceptual advantage” over tonal information and that the segmental information was available relatively earlier than the tonal information [5], [6], [7]. For example, in a speeded response discrimination task, Cutler & Chen (1997) asked Cantonese listeners to differentiate syllable pairs that differed in segmental (consonant and vowel) or supra-segmental (tone) aspects. They found faster and more accurate responses for the segmental than for the supra-segmental discriminations. In a tone-vowel detection task, Ye & Connine (1999) asked Mandarin speakers to detect whether some Mandarin syllables contained the vowel /a/ and the Tone 2 (e.g. ba2, lai2, yan2). They found that the detection time for vowel mismatches (e.g. bi2) was shorter than that for tonal mismatches (e.g., ba4). Moreover, it has been shown that Mandarin listeners were more interfered by the segmental than by the tonal information during a speeded classification task where they had to attend to one dimension of the stimuli (segmental or tonal) while ignoring the other dimension [8]. Overall, the studies indicate a tonal processing disadvantage as compared to the processing of segmental information in single words.

In contrast to the above-mentioned studies that showed relatively quicker access of segmental information as compared to tone information, Liu & Samuel (2007) argued that this was because the tasks used in these studies were sub-lexical in nature and that these tasks were performed without contextual constrains [9]. Word recognition generally occurs in context, which creates top-down expectations and might further influence lexical/phonological processing of the incoming words. Indeed, Ye & Connine [10] and Liu & Samuel [9] found a tone advantage in tone-vowel detection tasks when the target syllables occurred in highly constraining contexts using Mandarin (e.g. idiomatic phrases or sentential context).

Recently, an increasing number of studies used event-related potentials (ERPs) to investigate the role of tone and segmental information in lexical access in constraining context. The ERP technique is of particular advantage in the on-line study of speech processing, because it has high temporal resolution, and the measured ERP components reflect brain activities related to both perception and higher-order cognitive process of physical input without requiring complex task performance or the interruption of speech presentation for data collection. Two ERP components have been related to language processing. First, the N400 is a negativity that peaks around 400 ms after stimulus onset. The amplitude of the N400 is related to the semantic association between a word and its context, with larger N400s for the semantically less related words than for the more related words [11], [12]. Second, the late positive component (LPC) is a late centro-parietal positivity between around 500 ms and 1200 ms post-stimulus. It has been shown to be sensitive to syntactic violation and syntactic ambiguity [13], [14], [15], as well as semantic reverse anomalies [16], [17], [18].

Brown-Schmidt & Canseco-Gonzalez [19] studied the online processing of tonal and segmental information in Mandarin Chinese. They asked subjects to listen to normal and abnormal sentences in which the tone, the consonant, or both the tone and the consonant of the sentence’s final words were violated. They found that all the three types of anomalous words produced N400 effects compared to the normal words, with similar amplitudes and onset latencies. The results therefore suggest that the detection of tonal and segmental violations is essentially the same in time course. Likewise, Schirmer et al [20] manipulated the tone and vowel of the last words in Cantonese sentences. They found that the tone and vowel violations elicited similar N400 effects and LPC effects. Together, these studies demonstrate that listeners access tonal and segmental information at a similar speed and that these two kinds of information play comparable roles during speech processing. However, in the study by Hu et al [21], the authors asked subjects to judge the correctness of the last word of Mandarin idioms, which might deviate from the correct word in either tone or vowel. They found an early negativity only for vowel violation, and a stronger N400 effect for vowel violation than for tone violation. The authors thus concluded that vowels had an earlier and greater influence than tones on lexical selection and semantic integration of the spoken words into contexts.

Overall, it remains inconclusive whether the processing of tonal and segmental information differs in time during speech processing. Several reasons can be responsible for this. First, the studies using single syllables as stimuli did not necessarily ensure full lexical access of the words, because the tasks can be well completed based on sub-lexical knowledge. Second, since Mandarin is a tonal language, the tonal violation of comprehensible input (such as idioms and sentences) was inevitably confounded with a violation of semantic information. Finally, when the syllables were presented in highly constraining contexts, listeners inevitably generated strong expectations with regards to the meaning of the incoming words. In this sense, the processing of tonal and segmental information was strongly guided by the semantic expectation of words instead of their phonological analysis. Therefore, a new approach that offers insight into tone processing in constraining context with minimum semantic inference is needed in order to more directly investigate the phonological aspect of tonal and segmental information processing. In the present study, we intend to take advantage of the characteristics of classical Chinese poems to directly examine the roles of tones and vowels in speech processing.

**Figure 1:**
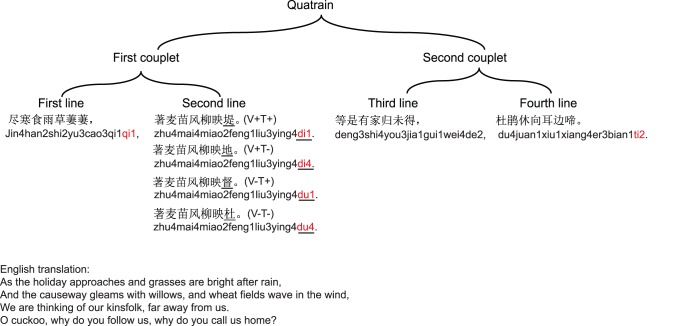
The hierarchical structure of a seven-character quatrain. There are four conditions for each experimental quatrain, which differ in the end of the last syllable of the first couplet (Line 2). The critical syllables are underlined. The Pinyin transcription is presented below each line of the quatrain. Correct: V+T+; Tone violation: V+T−; Vowel violation: V−T+; Combined violation: V−T−.

The characteristics of classical Chinese poems have primarily been described by Chinese linguists [22]. Classical Chinese poems have strict regulations with regards to meter (such as line length, the number of lines and syllables within a line) as well as rhythm (such as the presence of caesuras and tone contour). One form of classical Chinese poems includes a seven-character quatrain (so-called *Qijue*). Each quatrain consists of four lines (two couplets), with seven characters in each line. Moreover, each quatrain has a fixed rhyme (metrical feet). That is, the same primary vowel of the final sound appears repeatedly at the end of the first, second and fourth line (for example,/i/in the characters of “qi1”, “di1”, “ti2” in the end of the three lines in Figure 1). In addition, four tones are distinguished in Chinese language according to their acoustic properties: high level tone (tone 1), high rising tone (tone 2), falling-rising tone (tone3), and falling tone (tone4). These four tones are divided into “ping2” tone (meaning “flat”, including tone 1 and tone 2) and “ze4” tone (meaning “not flat”, including tone 3 and tone 4). In a Chinese poem, the “ping2” tone and “ze4”tone were generally presented alternately within a line, and the ending rhyme of the line always consists of a syllable with a “ping2” tone. Therefore, listeners have strong predictions regarding the phonological features (including both the tone and the primary vowel) of each syllable in the quatrain, while the exact upcoming character cannot be predicted because the meaning of the unknown quatrain is opaque to them. In this way, we can examine the processing of tones and vowels without confounds derived from semantic constraints. To the best of our knowledge, this is the first study to examine the perception of tones and vowels by using classical Chinese poems.

In the current study, we presented unfamiliar, seven-character quatrains to subjects aurally, with the last syllable in the second line of the quatrain either correct or deviating from the correct syllable at tone, vowel, or both levels. By examining the ERP responses to the different types of violations, we can determine the relative time course of tone and vowel processing. Since no semantic prediction can be generated from the unfamiliar quatrains, it remains an open question whether the N400/LPC effects will be observed as shown in previous studies [19], [20], [21]. It has been shown that the N450, starting at approximately 300 ms or earlier, and peaking around 450 ms over the midline and right hemisphere, is sensitive to rhyme, with a larger N450 elicited by non-rhyming than rhyming words [23]. Therefore, we expect an N450 effect for the vowel violation condition. However, no clear prediction is possible regarding the tone violation condition based on available data, because no ERP study has directly examined the tone processing in context in the absence of semantic expectation. Nevertheless, the effect latencies of the two types of violations are informative of the relative timing of tone and vowel processing.

## Materials and Methods

### Ethics Statement

The study was approved by the Institutional Review Board of the Institute of Psychology, Chinese Academy of Sciences. All participants provided written, informed consent before taking part in our experiment.

### Participants

Twenty right-handed students (mean age 23.2 years, range 20–25 years old, 8 males) took part in the experiment for payment. They were all native speakers of Mandarin Chinese without any visual or hearing disorders according to self-report.

### Stimuli

To ensure that all quatrains were unfamiliar and difficult to understand, we selected 240 quatrains out of a set of 300 quatrains based on a pretest. In the pretest, 16 participants who did not take part in the ERP experiment read the quatrains. They were required to first determine whether the quatrains were familiar to them (indicating Yes or No), and then to indicate the meaningness of these quatrains on a 7-point scale. We selected the quatrains that were unfamiliar (to which participants responded ‘No’ in the familiarity judgment task) and low in semantic meaningness (lower than 5 in the semantic meaningness score). The threshold of the meaningness was set at 5 as a result of balancing minimum semantic interference and enough quatrains to conduct the ERP experiment. The quatrains were presented visually in the pretest for the sake of efficiency. The semantic meaningness of quatrains presented in visual manner can be an overestimation because the meaning of the characters in a quatrain was comprehensible and subjects can comprehend the quatrains with no time limit. When the quatrains were presented auditorily, the unfamiliar quatrains became much more semantically meaningless due to frequency of homophones, complex syntactic structures of the quatrains, as well as limited time to listen to the quatrains. Therefore, we believe that the semantic meaningness of the quatrains was lower than 5 when they were presented in an auditory manner. The selection of unfamiliar and semantically meaningless quatrains ensured that the listeners could not actively predict what was coming based on their previous memory traces of the quatrains and that the processing of these quatrains can only be based on the perception of the quatrains’ rhythm instead of their meaning. Therefore, the brain responses to the quatrains largely reflected phonological processing rather than syntactic or semantic processing of the quatrains.

**Figure 2:**
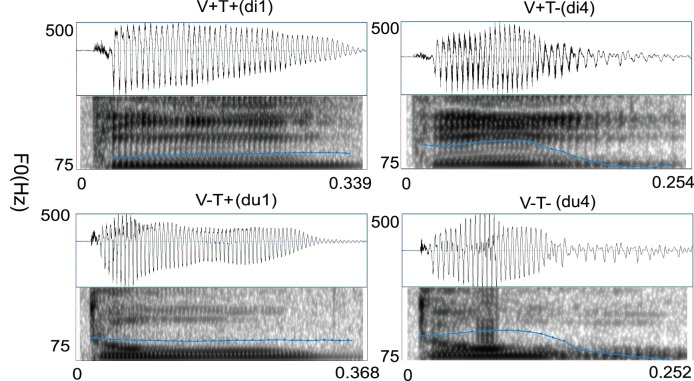
Acoustic features of the critical syllables in the four conditions of the example materials. Correct: V+T+; Tone violation: V+T−; Vowel violation: V−T+; Combined violation: V−T−.

In the ERPs experiment, we used 160 quatrains as experimental materials. For each quatrain, we manipulated the ending syllable of the first couplet by varying the lexical tone, vowel or both of them. This resulted in a full factorial design with four experimental conditions (see Figure 1): Correct (V+T+: correct vowel, correct tone), Tone violation (V+T−: correct vowel, incorrect tone), Vowel violation (V−T+: incorrect vowel, correct tone), and Combined violation (V−T−: incorrect vowel, incorrect tone). The tone violation was realized by using the tone 4 to replace the original tone 1 or tone 2 of the last syllable of the first couplet, while keeping the segmental information constant. We selected tone 4 as the violated tone because its pitch contour is significantly different from that of tone 1 and tone 2 (see Figure 2 for the pitch contour of the example materials). The vowel violation was realized by replacing one primary vowel with another one while keeping the tone and the total number of phonemes constant in the target syllable. Specifically, the original vowel (e.g., /*i/*) was replaced with another vowel (e.g., /*u/*) for the simple finals (e.g. /a/, /o/, /e/, /i/, /u/, /ü/), while the manipulation was applied to the whole final sound (e.g., replace /*an/* with /*ou/*) for compound finals (e.g., /*ai/, /an/, /ei/, /ou/*). The combined violation was realized by varying both the tone and the vowel of the correct condition. Therefore, a total of 640 quatrains were created. Experimental materials were grouped into four lists of 160 quatrains according to the Latin square procedure. In addition, 80 filler quatrains were also included in each list. The filler quatrains were comprised of 40 congruent quatrains and 40 incongruent quatrains. For the incongruent quatrains, the last syllable of the second couplet of the quatrains contained either tone violation (13 quatrains), vowel violation (13 quatrains), or combined violation (14 quatrains). The filler quatrains were included to prevent the participants predicting the specific position of violations. These fillers were excluded from data analysis. Overall, each participant in the present study listened to a set of 240 quatrains.

All the quatrains were read by a male expert speaker of mandarin Chinese and recorded at a sampling rate of 22 kHz. Since the tonal information normally coexists with segmental information [1], it can be difficult to separate them acoustically. Therefore, we only measured the duration of the whole target syllables. The duration of target syllables was on average 466 ms (SD = 73), 383 ms (SD = 53), 496 ms (SD = 75), and 391 ms (SD = 55) for the Correct, the Tone violation, the Vowel violation, and the Combined violation condition, respectively. Two-way repeated measures ANOVA were conducted with Vowel (correct, incorrect) and Tone (correct, incorrect) as independent factors, and the duration in each condition as the dependent factor. The statistical results revealed significant main effects of Tone and Vowel (*F*_(1,159)_ = 401.882, *p*<.0001; *F*_(1,159)_ = 38.321, *p*<.0001 respectively ), as well as a significant interaction between them (*F*_(1,159)_ = 14.623, *p*<.0001). Simple effect analysis showed that the duration of Tone correct conditions was longer than that of Tone incorrect conditions in both the Vowel correct (*F*_(1,159)_ = 233.24, *p*<.0001) and the Vowel incorrect conditions (*F*_(1,159)_ = 373.18, *p*<.0001), with a larger difference in the Vowel incorrect condition.

### Procedure

All the materials were presented aurally to the listeners in a pseudo randomized order in 8 blocks of 30 quatrains. The blocks were counterbalanced across runs. In each block, the same condition of quatrains was presented in no more than three consecutive trials. Each quatrain began with a fixation cross at the center of the monitor to minimize eye movements. Meanwhile, a warning tone “ding” was presented. After 300 ms, a quatrain was presented aurally, with the cross remaining on the screen. Participants were instructed to listen carefully and complete a “yes” or “no” judgment task on the appropriateness of the metrical feet after the presentation of each quatrain. The left and right hands to press the button were counterbalanced across different participants. Participants were also asked to avoid eye blinks and other body movements during acoustical presentation of the quatrains. The whole experiment lasted about two hours, including preparation, practice of five quatrains, and formal experiment.

### EEG Recordings

Participants were seated in front of a computer screen in an acoustically and electrically shielded room. The EEG was recorded using a 64-channel cap with Ag/AgCl electrodes, placed according to the international extended 10–20 system (passive electrodes). It should be noted that the choice of the reference settings has been shown to influence the observed ERP responses [24], [25]. The mastoid is the most common reference site in cognitive neuroscience and commonly used by investigators [26]. In order to make our study comparable to previous ERP studies [27], [28], [29], [30], the left mastoid served as reference during the recording, and the EEG data were re-referenced to the algebraic average of left and right mastoid electrodes. Vertical electrooculogram (EOG) was recorded in a bipolar montage by placing electrodes above and below the left eye, and horizontal EOG was recorded bipolary by placing electrodes at the outer canthus of each eye. The electrode impedance was lower than 5 KΩ. Recordings were done with a band pass filter of 0.05–100 Hz and a sampling rate of 500 Hz using AC amplifier.

### Data Analysis

The VEOG artifacts were automatically corrected by NeuroScan software 4.3 (for details of the method see [31]). We applied this correction procedure for ocular artifacts to keep enough number of trials in each condition. The data were then filtered with a low-pass filter of 30 Hz and were then segmented into epochs of −200 to 1500 ms relative to the onsets of critical syllable, with a baseline of 200 ms prior to the syllable onset. Then trials with amplitudes exceeding ±75 µV at any channel except for the EOG channels were removed. Trials with false responses were also removed. The remaining trials were on average 36, 37, 37 and 37 for Correct, Tone violation, Vowel violation and Combined violation condition, respectively. ERPs were computed for the critical syllables in each condition for each participant. Grand average ERPs were first visually inspected in order to identify time windows of interest. For the repeated measures ANOVA, we took the Vowel (correct, incorrect), Tone (correct, incorrect), Anteriority (frontal, central, parietal), and Hemisphere (left, middle, right) as independent factors, with the mean amplitude within the selected time windows and regions in each condition as dependent factor. Nine regions of interest (ROIs) were defined according to Anteriority and Hemisphere, with each having two or three representative electrodes: left frontal (F3, FC3, F5), left central (C3, CP3, C5), left parietal (P3, PO3, P5), midline frontal (FZ, FCZ), midline central (CZ, CPZ), and midline parietal (PZ, POZ), right frontal (F4, FC4, F6), right central (C4, CP4, C6), and right parietal (P4, PO4, P6). In cases in which the Tone or Vowel interacted with topographical factors, separate analyses were computed for different regions. The p values reported in the ERPs results section were adjusted with the Greenhouse–Geisser epsilon correction for nonsphericity. The uncorrected degrees of freedom were reported.

## Results

### Behavioral Results

**Figure 3:**
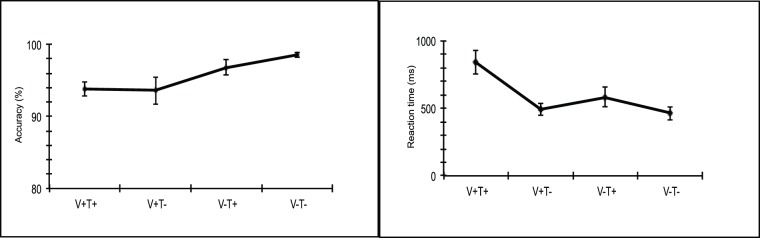
Reaction times and accuracy rates of the four conditions. Correct: V+T+; Tone violation: V+T−; Vowel violation: V−T+; Combined violation: V−T−. Error bars indicate the standard error (SE).

The behavioral results for all conditions are illustrated in Figure 3. ANOVAs treating Vowel (correct, incorrect) and Tone (correct, incorrect) as repeated-measures factors were conducted for accuracy and reaction time separately.

#### Accuracy

Participants were highly accurate in the rhyme judgment task (over 90% on average). ANOVA analysis for accuracy rate showed a significant main effect of Vowel (*F*_(1,19)_ = 10.02, *p = *.005), indicating that the judgment of the incorrect vowel conditions was more accurate than that of the correct vowel conditions. Both the main effect of Tone (*F*_(1,19)_ = .566, *p = *.461) and the interaction between Vowel and Tone (*F*_(1,19)_ = .975, *p = *.336) was not significant.

#### Reaction time

A repeated measures ANOVA for reaction time revealed main effects of both Tone (F_(1,19)_ = 23.16, p<.0001) and Vowel (F_(1,19)_ = 25.104, p<.0001), indicating that subjects’ responses towards correct conditions were slower than that of incorrect conditions. In addition, there was a significant interaction between Tone and Vowel(F_(1,19)_ = 11.329, p = .003). Simple effect analysis performed to test the vowel violation effect separately for the two tone conditions indicated that the subjects made slower responses for the correct vowel condition than for the incorrect vowel condition (F_(1,19)_ = 21.03, p <.0001) only when the tone was correct, whereas their responses were equally quick when the tone was incorrect (F_(1,19)_ = 1.09, p = .309). The RT showed different patterns than the duration of the stimuli, possibly due to the offline measurement of the RT.

### ERPs Results

Two time windows were selected for the statistical analysis based on visual inspection: 300–500 ms and 600–1000 ms. Only the effects containing the critical manipulations (Vowel violation and Tone violation) are reported.

#### 300–500 ms

**Figure 4:**
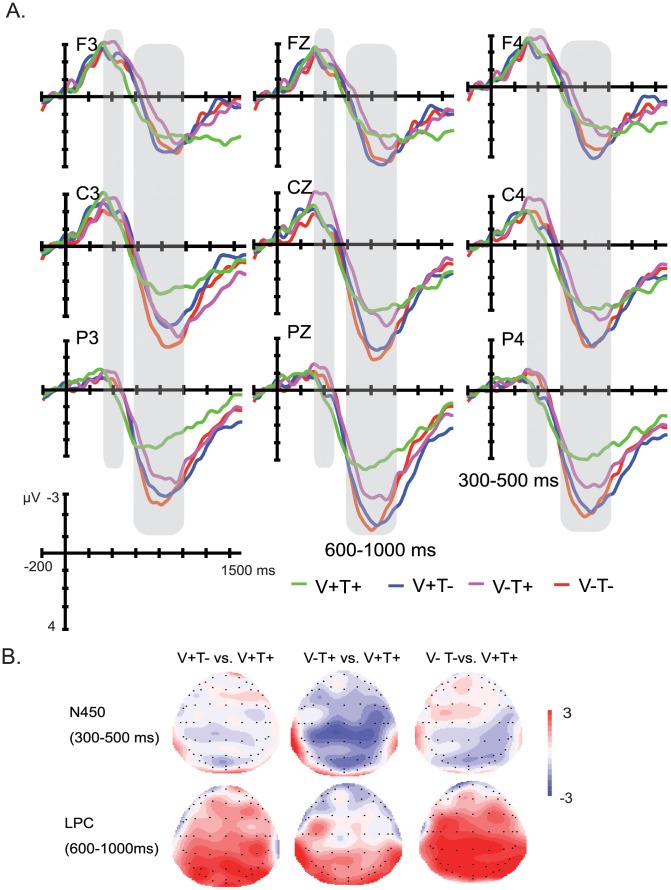
Grand average waveforms and topographical scalp distributions over 20 participants. (A) The waveforms are time locked to the onset of critical syllable in the end of the first couplet at selected channels of the four conditions. Negative is plotted upward. Correct: V+T+; Tone violation: V+T−; Vowel violation: V−T+; Combined violation: V−T−. (B) Topographical scalp distributions of the N450 and LPC effects evoked by different conditions.

Figure 4A shows the ERPs elicited by the critical words in the four conditions. The four-way ANOVA analysis indicated no significant main effect of Tone (*F*_(1,19)_ = .407, *p = *.531) or Vowel (*F*_(1,19)_ = 2.86, *p = *.107), neither was their interaction (*F*_(1,19)_ = 2.256, *p = *.15) significant. In addition, there was no significant interaction between Tone and Hemisphere (*F*_(2,38)_ = .954, *p = *.394), between Tone and Anteriority (*F*_(2,38)_ = 2.658, *p = *.107), between Tone, Hemisphere and Anteriority (*F*_(4,76)_ = .791, *p = *.534), between Vowel and Anteriority (*F*_(2,38)_ = 2.462, *p = *.129), between Vowel, Hemisphere and Anteriority (*F*_(4,76)_ = 1.545, *p = *.198), between Tone, Vowel and Hemisphere (*F*_(2,38)_ = .808, *p = *.453), between Tone, Vowel, Hemisphere and Anteriority (*F*_(4,76)_ = .723, *p = *.579). Although visual inspection seems to indicate an interaction between Vowel, Tone and Anteriority, no significant interaction effect was revealed from the statistical analysis (*F*_(2,38)_ = .833, *p = *.442). This might be caused by the large variability of the data in this time interval among the subjects. However, the interaction between Vowel and Hemisphere was significant (*F*_(2,38)_ = 4.878, *p = *.017). Simple effect analysis performed to test the Vowel violation effect in each region showed that the incorrect vowel conditions (as indicated by red and pink lines) elicited larger negativities than the correct vowel conditions (as indicated by green and blue lines) in the right hemisphere (*F*_(1,19)_ = 6.05, *p = *.024), but not in the left hemisphere (*F*_(1,19)_ = .67, *p = *.425) and the midline (*F*_(1,19)_ = 2.67, *p = *.119).

#### 600–1000 ms

We found that the incorrect tone conditions (as indicated by red and blue lines) elicited larger positivities than the correct tone conditions (as indicated by green and pink lines), which was evidenced by the main effect of Tone (*F*_(1,19)_ = 9.028, *p = *.007). There was also a marginally not significant interaction between Tone and Anteriority (*F*_(1,19)_ = 3.601, *p = *.056). Simple effect analysis indicated that the effect of Tone violation was located in central (*F*_(1,19)_ = 7.8, *p = *.012) and parietal areas (*F*_(1,19)_ = 22.49, *p*<.0001), but not in frontal areas (*F*_(1,19)_ = 2.07, *p = *.167). Although the main effect of Vowel was not significant (*F*_(1,19)_ = 1.769, *p = *.199), an interaction between Vowel and Anteriority was found (*F*_(2,38)_ = 7.856, *p = *.007). Simple effect analysis indicated that the incorrect vowel conditions elicited larger positivities than the correct vowel conditions mainly over posterior areas (*F*_(1,19)_ = 7.01, *p = *.016), but not frontal (*F*_(1,19)_ = .01, *p = *.918) and central areas (*F*_(1,19)_ = 1.94, *p = *.18). Furthermore, the interaction between Tone, Vowel and Anteriority was also significant (*F*_(2,38)_ = 5.931, *p = *.014). Simple effect analysis performed to test the Vowel violation effect in each region indicated that when the tone was correct, the incorrect vowel conditions elicited larger positivities than the correct vowel conditions over parietal areas (*F*_(1,19)_ = 6.34, *p = *.021) but not frontal (*F*_(1,19)_ = .1, *p = *.757) and central areas (*F*_(1,19)_ = .58, *p = *.454). However, when the tone was not correct, there was no significant difference between correct and incorrect Vowel conditions in frontal (*F*_(1,19)_ = .22, *p = *.643), central (*F*_(1,19)_ = 2.18, *p = *.156) and parietal areas (*F*_(1,19)_ = .5, *p = *.487). In addition, there was no significant interaction between Tone and Hemisphere (*F*_(2,38)_ = 1.301, *p = *.284), between Tone, Hemisphere and Anteriority (*F*_(4,76)_ = 1.016, *p = *.404), between Vowel and Hemisphere (*F*_(2,38)_ = .881, *p = *.423), between Vowel, Hemisphere and Anteriority (*F*_(4,76)_ = 1.14, *p = *.344), between Tone and Vowel (*F*_(1,19)_ = .091, *p = *.767), between Tone, Vowel and Hemisphere (*F*_(2,38)_ = .04, *p = *.961), between Tone, Vowel, Hemisphere and Anteriority (*F*_(4,76)_ = 2.139, *p = *.101).

Overall, the incorrect vowel conditions elicited larger negativities than the correct vowel conditions over the right hemisphere, while the incorrect tone conditions elicited larger positivities than the correct tone conditions over central and parietal regions. In addition, the incorrect vowel condition elicited larger positivities than the correct vowel condition over posterior regions when the tone was correct, whereas the incorrect and correct vowel conditions elicited equally large positivities when the tone was incorrect (Figure 4B).

## Discussion

In the present study, we used seven-character quatrains to investigate the processing of tone and vowel information during speech processing. At the behavioral level, subjects made quicker and more accurate responses to the incorrect quatrains (either tone or vowel violation) than the correct quatrains. At the electrophysiological level, the incorrect vowel conditions elicited larger negativities (in the time window of 300–500 ms) than the correct vowel conditions over the right hemisphere regardless of the tone correctness. In the later time window (600–1000 ms), the tone violation conditions elicited larger positivities than the tone correct conditions over central and posterior regions. Moreover, we found an interaction between the effect of tone and vowel violations over posterior region, showing a larger positivity for the incorrect vowel condition than for the correct vowel condition when the tone was correct.

### The Early Negative Effect in Response to Vowel Violation

We found that the violated vowels elicited more negative-going waves than the correct vowels within the 300–500 ms time window over the right hemisphere. Previous studies using intelligible sentences as stimuli reported N400 effects in response to tone and vowel violations [19], [20], [21]. Since the present effect was also caused by vowel violation and fell in the classical N400 time window, one might take it as an N400 effect. However, this is unlikely for the following reason. The N400 has been related to lexical-semantic retrieval or semantic integration of words into context [11], and the N400 effects found in previous studies [19], [20], [21] all involved lexical-semantic processing. In the current study, however, the unfamiliar quatrains were not high in meaningness, and thus the activated lexical-semantic should be very limited. Another ERP component that occurs in a similar time window is the N200 component. It has been related to detection of novelty or mismatches [32] and has been taken as a reflection of cognitive control. However, the N200 effect elicited by auditory stimuli typically has a frontal-central scalp distribution, which differed from the right (posterior) distribution in the current study. Moreover, both the vowel and the tone violations would have elicited such an N200 effect because this effect has been shown to be sensitive to detection of mismatch in general. Therefore, the present negative effect might not be an N200 effect.

Given its eliciting condition, wave morphology and temporal characteristics, we took the negative effect as an N450 effect [23], [27], [28], [33], [34]. The N450 effect was found to be elicited by comparing rhyming and non-rhyming stimuli, including isolated letters [23], [29], [30], multi-syllabic pseudowords [35], and nonwords [23], [28]. It has been hypothesized that subjects generated a set of candidate words that were likely to match with the first word during the interstimulus interval between the prime and the target in a rhyme-matching task, and the enhanced N450 was induced when the target did not belong to the set of candidates [34]. In the current study, we used quatrains to create strong constraints towards the tonal and vowel characteristics of the final syllable in the first couplet. The N450 rhyme effect reflects that subjects actively predicted the vowel of incoming syllable even when they did not access the lexical-semantic information of the input. As far as we know, although the N450 effect has been found in Dutch [22], [36], French [29], [37], [38], and English [23], [28], [29], [30], [33], [34], this is the first time to detect this effect in classic Chinese poem. Furthermore, the right hemisphere distributed N450 rhyming effect is generally consistent with the scalp distribution of previous studies [23], [28], [30], [33], [34], which is reported to be prominent over the midline and right hemisphere. Overall, we have examined phonological processing while strictly controlling lexical-semantic processing using unfamiliar quatrains. This constitutes the unique contribution of the current study.

Unlike the vowel violation, we found no N450 effect for the tone violation (e.g., *di1– di4*). This result clearly indicates the processing difference between tone and vowel. In contrast to the view that the N450 is sensitive to phonological overlap in general [36], [37], [38], the lack of an N450 effect for the tone violation in the present study suggests that the N450 is specifically sensitive to the vowel rhyming instead of general phonological similarities. The specification of the N450 towards rhyming is in line with another study where word pairs that shared a phonological overlap but did not rhyme (e.g., *bell – ball*) elicited no N450 effect [23].

### The Late Positive Effect in Response to both Vowel and Tone Violations

In the later time window (600–1000 ms), we found larger positivities for both the tone and the vowel violation conditions compared to the correct condition, with an interaction between them over posterior region. We defined this positive shift as an LPC based on previous studies [16], [21], [39]. The LPC effect evoked by tone or vowel violation has also been found in other studies [20], [21]. In language studies, the LPC effect has been evoked by syntactic violation, syntactic ambiguity, as well as semantic anomaly [40], [41], [42], [43]. Some researchers have associated it with error detection [44] or reanalysis processing [16], [39]. In the present study, both the tone and vowel violations elicited the LPC effects. The vowel violation only elicited an LPC effect when the tone was correct, whereas the tone violation elicited an LPC effect regardless of the vowel’s correctness. As mentioned above, the vowel violation has evoked an N450 effect at the early stage, so the LPC effect yielded by the tone violation indicates the relatively delayed detection of the tone violation compared to the vowel violation. This finding is particularly informative in terms of the acoustic features of vowel and tone in Chinese syllable. Whereas vowel belongs to segmental information, tone belongs to supra-segmental information. That is to say, the realization of tonal variation is generally concurrent with vowel articulation [45], so it is difficult to separate the time course of tone from that of vowel in phonetic analysis. Using ERPs, we were able to dissociate the differential processing of tone and vowel. The finding is consistent with the behavioral findings in single word processing [5], [6], [7], which indicated that segmental information was available relatively earlier than the tonal information. However, to the best of our knowledge, this is the first study that distinguishes the time course of vowel and tone processing at the sentence level with limited activation of lexical-semantic information. In addition to tone violation, vowel violation also elicited an LPC effect when the tonal information was correct. Since the vowel violation has been detected earlier (as indicated by the N450 effect), the LPC effect in response to the vowel violation might indicate the reanalysis of vowel information when the tone was not violated. No such reanalysis was necessary when tone violation also occurred, so no LPC effect was elicited by vowel violation in the tone incorrect conditions. The interaction between the effect of tone and vowel violations at this processing stage (600–1000 ms) indicates that the detection of tone violation co-occurred with the reanalysis of vowel information. Therefore, in line with previous studies, the LPC effect reflects both error detection and reanalysis of the input [16], [39], [44]. Moreover, the LPC effect evoked by tone and vowel violations in the current study indicate that the LPC effect is not solely sensitive to syntactic or semantic processing, but also to phonological processing.

It is noteworthy that although the present study used a delayed response task, the LPC results showed a similar pattern as the RT data: the higher amplitude of the LPC, the shorter time the subjects spent to make a response. This may be caused by the following reason. When the subjects perceived a violation in the middle of a quatrain, they were ready to make an “inappropriate” response before the whole quatrain ended. However, they would need to wait till the end of the correct quatrain in order to make an “appropriate” response. Therefore, the violating conditions elicited larger LPCs and shorter RTs. Nevertheless, as an offline measurement, the RT data reflect a combination of several aspects of processing (e.g., perception, decision and motor operations), so the relationship between the RT data and the LPC amplitudes might be complicated. It will be interesting to further explore the underlying relationship between the RT and the ERP responses. Moreover, there was no correspondence between the ERP effects and the duration of the target syllables, indicating dissociation between the acoustic properties and the perception of the syllables.

### Implications to Speech Perception Model

One might wonder whether the present results can be generalized to everyday Chinese speech. Although the phonological characteristics of the Chinese syllables are the same between poem and everyday speech, the poem has a much stronger phonological constraint than Chinese everyday speech. Therefore, it remains to be seen whether similar findings can be obtained with everyday Chinese. Similarly, it requires future studies to determine whether our findings can be extended to Indo-European languages, which also contain segmental and supra-segmental information (such as pitch accent in English and Dutch). The perceptual advantage of segmental (e.g., vowel) over supra-segmental (e.g., tone) information is consistent with speech perception model, such as the TRACE model [46]. The model was constructed by three hierarchical levels, namely feature (i.e., an approximation of acoustic spectra extended in time), phoneme, and lexical nodes. These levels interact with each other via excitatory and inhibitory connections, and perform in a dynamic fashion during speech perception. The excitatory connection from lexical to phoneme nodes permits contextual information to exert influence to their activation. On the basis of this model, Ye and Connine (1999) added a separate tonal level “toneme” to indicate that tones have a similar status to phoneme in the modified version of this model [10]. They assumed that the degree of activation for both toneme and phoneme was based on the quality of the input signal and the lexical feedback connection. In the absence of any context, it has been indicated that tone identification normally involves the processing of F0 and is primarily realized in vowels. Therefore, the tone information cannot be processed until the vowel information is available [6]. As a result, vowel perceptive advantage can be found in single word levels [5], [6], [7]. When the syllables were presented in highly constraining sentence context as conducted in previous studies [19], [20], both tone and vowel benefit from the robust lexical activation from sentence context. The strong activation of lexicon-toneme can offset the perceptive disadvantage of tone, and thus a similar access time for vowel and tonal information resulted. In the present study, although the poem context can constrain the acoustic realizations of tone and vowel of the target syllable, there is no robust lexical activation to generate a unique syllable. In this sense, both toneme and phoneme (vowel) received little (if any) feedback from the lexical code, and thus they can mainly be activated via the feed forward activation from features of acoustic input as well as the phoneme-toneme connections. Therefore, a vowel advantage was found when the perception occurred in phonologically constrained contexts. Our results, in support of the TRACE model, indicate the dynamic interactions between and within different levels (i.e., feature, phoneme, lexical) of speech analysis during speech perception.

## Conclusions

We examined the time course of vowel and tone processing in classical Chinese poems, where the lexical-semantic information was not necessarily activated. While the detection of vowel violation elicited an N450 effect, the detection of tone violation yielded an LPC effect. In addition, there was an interaction between the effect of vowel and tone violations during the LPC time window, showing an LPC effect for the vowel violation only when the tone was correct. Overall, the results suggest that people process the vowel information (around 300–500 ms) prior to that of the tone information (around 600–1000 ms), and that people integrate both the vowel and tone information in the later processing stage (600–1000 ms).
